# Research on Collision Access Method for Satellite Internet of Things Based on Bayliss Window Function

**DOI:** 10.3390/s25103112

**Published:** 2025-05-14

**Authors:** Xinjie Zhao, Ziwei Liu, Yuanyuan Xu, Yihan Du, Bin Lyu, Leiyao Liao, Gengxin Zhang

**Affiliations:** School of Communications and Information Engineering, Nanjing University of Posts and Telecommunications, Nanjing 210003, China; 2022010108@njupt.edu.cn (X.Z.); 1221014535@njupt.edu.cn (Y.X.); duyihan271@outlook.com (Y.D.); blyu@njupt.edu.cn (B.L.); 20230117@njupt.edu.cn (L.L.); zgx@njupt.edu.cn (G.Z.)

**Keywords:** beamforming, random access, satellite Internet of Things, signal separation

## Abstract

Satellite Internet of Things (IoT) terminals face design constraints regarding low power consumption and light control. These constraints pose a significant collision risk when utilizing traditional random-access protocols, making it challenging to meet the system throughput requirements. Auxiliary beam schemes based on conventional beam formation suffer from the problem of the auxiliary beam shape being limited by the fixed directional map. This leads to the problem of limited throughput enhancement. In this paper, an auxiliary beam weight optimization method for satellite IoT capacity enhancement is proposed. By increasing the number of main flap roll-off bands, the success rate of collision signal separation is increased. It is possible to improve the system access performance. The simulation results indicate that the proposed method can significantly improve the system throughput performance. Furthermore, it can withstand some direction of arrival (DOA) estimation errors and amplitude–phase errors. Robustness is possessed.

## 1. Introduction

With recent advancements, the Internet of Things (IoT) has permeated various facets of societal life. The conventional terrestrial IoT network based on WiFi, narrowband IoT, and LoRa access technology adequately fulfills communication requirements in urban areas and normal workplaces [1]. However, areas such as deserts, oceans, and forests have lower population densities. The terrestrial IoT network based on the terrestrial cellular network cannot be constructed due to geographical factors and economic cost constraints [2]. In this case, the low Earth orbit (LEO) satellite communication system offers seamless global coverage and high resilience to damage. It can address the limitations of conventional ground-based IoT coverage. Therefore, the LEO satellite communication system is an important component of the future 6G integration of sky, earth, and sea for intelligent connectivity of all things [3,4].

In the satellite IoT scenario, communication networks are becoming more intricate, and new business models are consistently emerging. A growing number of heterogeneous terminals with diverse service demands are expected to access satellite networks. However, terminals transmit short-burst data packets with minimal control overhead and low power consumption, resulting in frequent collisions in massive access scenarios. Consequently, the utilization rate of satellite system resources decreases, and the capacity increase is limited. Therefore, enhancing terminal access success rates to improve system throughput has become a critical research focus. Existing studies on random-access techniques can be broadly classified into three categories. The first type of random-access protocol is known as the traditional random-access (ALOHA) protocol. The second type of random-access protocol makes use of interference cancellation techniques and packet transmission diversity. The third type of random-access protocol introduces dimensions such as code domain, polarization domain, and power domain.

ALOHA protocol [5] sends data immediately as soon as the terminal has it. Although it has a low throughput and is prone to collisions, it has the least complex implementation. On this basis, Roberts divides the transmission time into multiple time slots. The Slotted ALOHA (SA) protocol was proposed in the literature [6]. The data transmission of this protocol is based on time-division multiple access (TDMA) frames. Terminal data can only be sent at the beginning of each time slot. This method reduces the randomness of the terminal sending data packets. Thus, the probability of conflict can be reduced to improve system throughput. However, when multiple terminals transmit data packets within the same time slot, collisions may still occur, leading to transmission failures. In order to improve system throughput, random-access protocols utilizing packet transmission diversity and interference cancellation techniques were proposed. The literature [7,8,9,10,11] proposed such RA methods based on competitive resolution diversity slots as Diversity Slotted ALOHA (DSA), Content Resolution Diversity Slotted ALOHA (CRDSA), CRDSA++, Irregular Repetition Slotted ALOHA (IRSA), Coded Slotted ALOHA (CSA), etc. These schemes employ the time diversity characteristics, which improve the system throughput to varying degrees. Nevertheless, this method still has some drawbacks. Firstly, the user terminal sends replicas of the packet across various slots within the frame, which notably raises the terminal’s power usage for transmission and reduces the system’s energy efficiency. Moreover, since most of these methods depend on clean copies, the packets that are free from interference can be recovered. Secondly, the separation process cannot be triggered at medium and high loads since the receiving condition of collision tolerance is a collision-free slot. In the face of massive terminal access, the system throughput drops sharply under medium and high loads. Accordingly, it is still not fully applicable to the satellite IoT massive terminal access scenario. In the third type of RA protocol, the time domain, polarization domain, and power domain dimensions are introduced to improve the competition space and improve system throughput performance. Reference [12] proposed Non-Orthogonal Slotted ALOHA (NOSA) based on the time domain. Reference [13] introduced the polarization domain and proposed a Polarized Multiple-Input Multiple-Output Slotted ALOHA (PMSA) protocol combined with power diversity. Non-Orthogonal Multiple Access (NOMA) technology, as a new type of multiple access method, allows different users to reuse resources in the time–frequency domain. NOMA has high spectral efficiency and good fairness. The receiving end uses Iterative Serial Interference Cancellation (SIC) technology to achieve correct demodulation of user signals [14]. This method can improve system throughput to support massive connections in satellite IoT. Currently, the realizability of power domain signal separation is strong. Utilizing the power domain to separate collision signals stands out as a highly promising solution [15,16,17]. The literature [18,19] introduced the power dimension CRDSA protocol. This protocol obtains the difference in received signal power by utilizing transmission power diversity or natural fading of the channel. Furthermore, the capture effect is utilized at the receiving end to enhance system throughput. The non-orthogonal multiple access IRSA (NOMA-IRSA) protocol, in combination with power diversity, was suggested in the literature [20,21].

To address collision-induced low throughput and “avalanche effects” in satellite IoT systems with negligible near–far effects and no power control, the previous study proposed an auxiliary beam design using conventional beamforming [22]. By dynamically adjusting the auxiliary beam’s pointing direction based on collision packet DOA and exploiting roll-off bands to create power gain differentials, the method enhanced signal separation, achieving superior throughput and robustness over traditional random-access schemes. However, unlike conventional satellite receiving beams, the auxiliary beam retains a fixed shape, with only its orientation being adjusted. The difference in gain for receiving collision signals is achieved by utilizing the roll-off band of the auxiliary beam. This approach offers low complexity. Nevertheless, due to the constraints imposed by the fixed beam patterns on auxiliary beams, there is a limitation on the enhancement in system throughput.

Aiming to address the issues mentioned above, an auxiliary beam design scheme based on Bayliss window function weight optimization is put forward. By increasing the number of main flap roll-off bands, the collision signal separation success rate can be increased. Therefore, the system access performance is improved. Specifically, this scheme utilizes the Bayliss weighting method to generate differential beams. Subsequently, this differential beam is employed to generate a supplementary beam, leading to zero sags at the center of the main flap. The power gain disparity of colliding signals is further amplified. The scheme proposed in this paper further improves the success rate of collision signal separation based on the auxiliary beam pointing scheme. As a result, the system access performance can be improved, which can effectively support the large-capacity random-access requirements of satellite IoT terminals.

The main contributions of this paper are summarized as follows:

▪ To overcome throughput limitations imposed by fixed auxiliary beam patterns, we propose a novel auxiliary beam design scheme utilizing Bayliss window function-based optimization. This scheme generates a main lobe with a central null and precisely controlled roll-off regions to enhance power gain disparities between colliding signals.
▪ Simulations validate the superiority of the proposed scheme over existing methods, demonstrating a 108% improvement in collision resolution success rate compared to prior auxiliary beam adjustments. Moreover, the proposed scheme exhibits strong robustness, achieving a 28% peak throughput improvement by combining angle and amplitude–phase errors.

The remaining content of this paper is structured as follows. In Section 2, the system model is introduced. A collision-tolerant access method based on auxiliary beams is provided in Section 3. Section 4 presents the simulation results and analysis of the throughput and packet loss performance of this method. Finally, in Section 5, the full text is summarized, and the conclusions are given.

## 2. System Model

### 2.1. Signal Access Scenario

**Figure 1:**
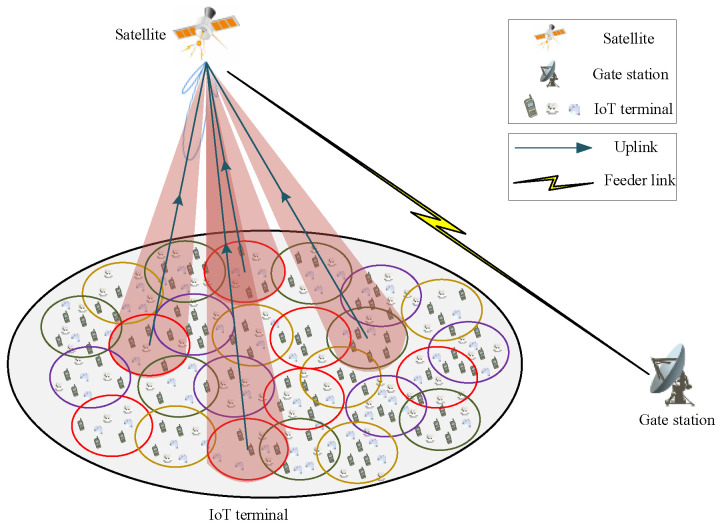
Uplink RA scenario for satellite IoT packets.

Considering the scenario of random uplink access for LEO satellite IoT user links, the satellite beam adopts a spatial coverage method of multi-beam and multi-color multiplexing. Each of these beams employs the phased array conventional beamforming method, with adjacent beams operating on distinct frequencies to prevent signal interference. This design enables a single communication beam of the satellite to concurrently cover numerous terminals on a large scale. The access mode adopts the traditional SA protocol in the RA protocol. Multiple active terminals on the ground randomly select different time slots in the same access frame to send data packets to the satellite. Figure 1 shows the uplink RA scenario for satellite IoT packets.

### 2.2. System Access Model

The spatial domain received signal model is considered a uniform linear array (ULA) with N array elements. Suppose that each element is isotropic. The spacing between elements is d. It is assumed that all the P+1 spatially incident signals are far-field narrow-band signals. $w_{1},w_{2},\ldots ,w_{N}$ are the weighted values of the received signals of each array element channel, respectively. The array pattern is defined as the array response of a given array weight vector W to signals at different angles. The first array element on the left is taken as the reference array element. Considering the magnitude and phase errors, the array response is denoted as

(1) $$F(\theta )=W^{H}\tilde{a}(\theta ),$$

(2) $$W={\left[w_{1},w_{2},\ldots ,w_{N}\right]}^{T},$$

(3) $$\tilde{a}(\theta )=\Gamma \Phi a(\theta ),$$

where $\Gamma =diag\left(\rho_{1},\rho_{2},\ldots ,\rho_{N}\right)$, $\Phi =diag\left(e^{j\pi \varphi_{1}},e^{j\pi \varphi_{2}},\ldots ,e^{j\pi \varphi_{N}}\right)$, $\rho_{j}$ is the amplitude gain of the jth element, and $\varphi_{j}$ is the phase gain of the jth element. Moreover, $\rho_{1}=1,\;\mathrm{and}\;\varphi_{1}=0$. Therefore, the array response with amplitude and phase errors is represented as

(4) $$F(\theta )=W^{H}\Gamma \Phi a(\theta ).$$

### 2.3. Overall Process

**Figure 2:**
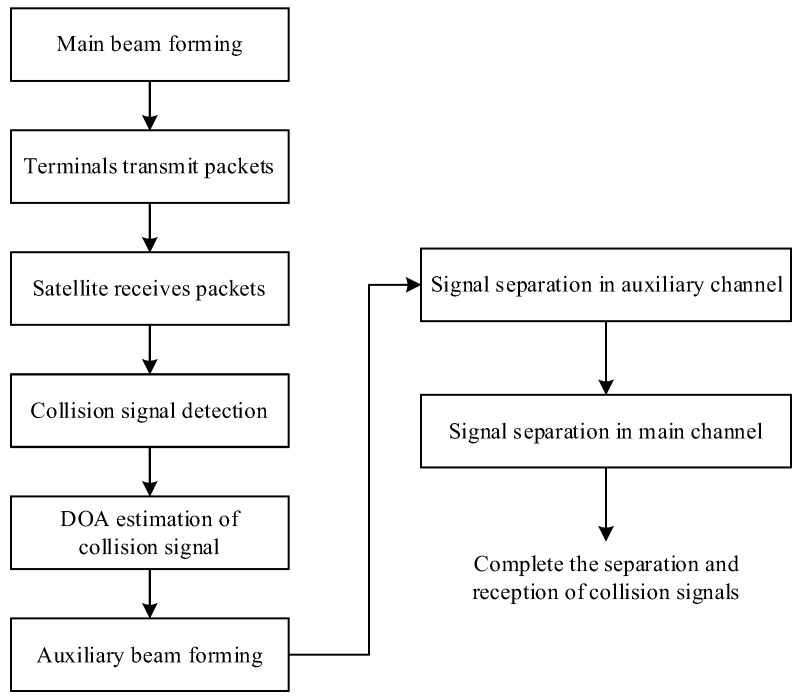
Collision packet separation process.

Considering the coverage and transmission requirements of the business beam, the method proposed in this paper does not alter the direction and orientation of the conventional receiving beam, which is referred to as the main beam in the following text. In this paper, the main beam is coupled with an auxiliary beam. The additional auxiliary beam is formed on the basis of the main beam. Moreover, the auxiliary beam and the main beam cover the same area and work simultaneously. Within the 3 dB beamwidth range of the main beam, the gain of collision signals in the main beam on the auxiliary beam is optimized and designed. Indirectly, the collision signal power difference is obtained. The separation process is shown in Figure 2.

In the above process, collision signal detection can adopt methods based on pilot signals to detect and capture short burst signals, such as frequency domain detection method [23], power detection method [24], etc. DOA estimation can be achieved using DOA estimation based on deep convolutional neural networks [25], MUSIC algorithm based on eigenvalue clustering [26], and other methods. Signal separation adopts SIC technology. The SIC algorithm recursively improves symbol estimation and eliminates residual interference, effectively subtracting the estimation of multi-user interference from the received signal. This paper focuses on the formation of auxiliary beams under the assumption that the DOA of collision signals is known.

## 3. Methods

**Figure 3:**
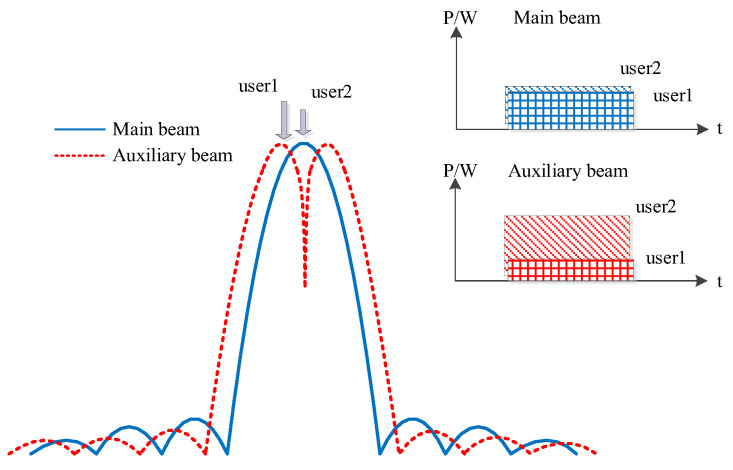
Illustration of auxiliary beam design for generating differential beams based on Bayliss weighting method.

By constructing an auxiliary beam, the auxiliary channel is set to rapidly decrease in gain near the center of the main beam. The power gain difference between the peak–valley values of the main lobe of the auxiliary beam is used to extend the power difference in the collision signal. The key to designing an auxiliary beam lies in solving the null position and gain variation within the main lobe of the auxiliary beam under different collision signal arrival directions and different collision weights. An illustration of the auxiliary beamforming is shown in Figure 3. Users within the main beam exhibit comparable power levels, while power differentiation between users is primarily established through auxiliary beamforming.

### 3.1. Basic Principle of Bayliss Weighting Method

Based on the above analysis, the differential beamforming method is considered for generating auxiliary beams. The differential beamforming is to form a differential beam at the output end of the receiving system through the common beamforming method and form a null in a specified direction. In this paper, the Bayliss weighting method is adopted to generate the differential beam. By adding windows to the guiding vector in the direction of the beam, a differential beam that satisfies the conditions is designed. The traditional differential beam window is the Bayliss window. The Bayliss distribution is a typical difference distribution, which makes the phases of the left and right elements of the array invert each other. The weighted Bayliss differential beam directional diagram is described by two parameters, which control sidelobe levels and sidelobe envelope attenuation characteristics. The weight of the Bayliss window function is expressed as

(5) $$W_{Bayliss}(p)=\sum_{m=1}^{\bar{n}-1}B_{m}\sin[\pi (n+1/2)(\frac{2p-N-1}{N-1})],1\leq p\leq N,$$

where $B_{m}$, σ, and $z_{n}$ are represented by Equations (6), (7) and (8), respectively.

(6) $$B_{m}=\left\{\begin{array}{l} \frac{1}{2j}{\left(-1\right)}^{m}{\left(m+1/2\right)}^{2}\frac{\prod_{n=1}^{\bar{n}-1}\left\{1-\frac{{\left[m+1/2\right]}^{2}}{{\left[\sigma z_{n}\right]}^{2}}\right\}}{\prod_{\begin{array}{l} n=1\\ n\neq m \end{array}}^{\bar{n}-1}\left\{1-\frac{{\left[m+1/2\right]}^{2}}{{\left[n+1/2\right]}^{2}}\right\}},m=0,1,2,\ldots \bar{n}-1\\ \;0,\;m\geq \bar{n} \end{array}\right.,$$

(7) $$\sigma =\frac{\bar{n}+1/2}{z_{\bar{n}}},z_{\bar{n}}={\left({A_{n}}^{2}+{\bar{n}}^{2}\right)}^{1/2},$$

(8) $$z_{n}=\left\{\begin{array}{ll} 0, & n=0\\ \pm \Omega_{n}, & n=1,2,3,4\\ \pm {\left(A^{2}+{\bar{n}}^{2}\right)}^{1/2}, & n=5,6,\ldots \end{array}\right.,$$

where $\bar{n}$ is the number of sidelobe adjacent to the main lobe of the expected constraint, N is the number of matrix elements, and R is the sidelobe level. The coefficients A and $\Omega_{n}$ cannot be expressed in closed form. Bayliss listed a fourth-order polynomial coefficient, as shown in Table 1.

The calculation formula of A and $\Omega_{n}$ is expressed as Bayliss

(9) $$A\;or\;\Omega_{n}=\sum_{n=0}^{4}C_{n}{\left(-R\right)}^{n}.$$

The polynomial coefficient $C_{n}$ in the formula is shown in Table 2.

The output of the array is indicated by the following formula:

(10) $$\begin{array}{ll} F & =W_{a2}^{H}\tilde{a}\left(\theta \right)\\ & =\lambda \cdot {\left(W_{a2}\cdot W_{Bayliss}^{H}(\bar{n},R,N)\right)}^{H}\Gamma \Phi a\left(\theta \right)\\ & =\lambda \cdot {\left(a\left(\theta_{a2}\right)\cdot W_{Bayliss}^{H}(\bar{n},R,N)\right)}^{H}\Gamma \Phi a\left(\theta \right), \end{array}$$

where the auxiliary beam weight vector is represented by $W_{a2}=a\left(\theta_{a2}\right)$, and $W_{Bayliss}$ is the Bayliss window function weight. $\theta_{a2}$ is the direction of the auxiliary beam, a2 represents the optimization of the auxiliary beam design scheme based on the weight of the Bayliss window function, and λ is the amplitude gain coefficient of the auxiliary beam.

The power gain of the receiving antenna for the auxiliary beam is expressed as

(11) $$G_{ra2}\left(\theta ,\theta_{a2},\lambda ,R\right)={\left|\lambda \cdot {\left(a\left(\theta_{a2}\right)\cdot W_{Bayliss}^{H}(\bar{n},R,N)\right)}^{H}\Gamma \Phi a\left(\theta \right)\right|}^{2}.$$

An analysis is conducted on the conditions of collision separation in the power domain. The weaker power signal in the collision signal can be regarded as the interference in the demodulation of the stronger power signal. The interference signal power of the ith strong signal is expressed as $I=\sum_{j>i}C_{j}$. It can be concluded that the objective of the optimization problem is to maximize the carrier–dry-noise ratio of the collision signal to determine whether the collision signal can be separated. If the maximum carrier-to-noise ratio of the collision signal exceeds the separation threshold, it indicates that the separation can be achieved. Since we adjust the separation power difference in the auxiliary beam, the weakest signal can be tolerated below the demodulation threshold in the auxiliary beam. Afterward, through the step cancellation between the main and auxiliary beams, the main beam can still meet all the successful reception. Since the collision signals in the auxiliary beam cannot all be lower than the demodulation threshold, the angle difference between the two basic beams’ directions and the main beam direction should be limited to the main lobe width of the main beam.

### 3.2. Establishment of Optimization Model

In the time-slot ALOHA system, the number of packets colliding in each time slot is different due to different loads. In this paper, on the basis of ensuring the maximum number of collision signals successfully separated from each time slot, we study the serial multiple iterative optimization method by time slot. According to the above analysis of the principle of the Bayliss weighting method for generating differential beams, only three parameters of the weight function are controllable: sidelobe level R, equal sidelobe level $\bar{n}$ and array element N. The number of sidelobes $\bar{n}$ adjacent to the main lobe of the expected constraint is generally taken as 4 or 5. Moreover, the gain of the main flap of the differential beam generated by this scheme is reduced compared with that of the main beam. Therefore, parameter R and amplitude gain coefficient λ are used as variables for differential beam optimization. Assume that $H_{m}$ packets collide in the m time slot. The mathematical model based on differential beam separation for multiple collision data packets is established as follows

(12) $$\begin{array}{l} \;\underset{R,\lambda }{\max}\frac{C_{i}}{\sum_{\begin{array}{l} j>i\\ i\neq H_{m} \end{array}}C_{j}+N_{a}}.\\ s.t.\;C_{1}:\frac{C_{i}}{\sum_{\begin{array}{l} j>i\\ i\neq H_{m} \end{array}}C_{j}+N_{a}}>\delta \\ \;C_{2}:0<\lambda <\kappa \;(i<j,i\sim j\in Q),\\ \;C_{3}:\varepsilon_{1}<R<\varepsilon_{2} \end{array}$$

where $C_{i}$ and $C_{j}$ are the carrier powers ($C_{i}>C_{j},i<j$) of the collision signals, $N_{a}$ is the equivalent noise power, and Q is the set of collision signal pair numbers. δ is the separation threshold, λ is the auxiliary beam amplitude gain coefficient, and κ is the upper limit of the amplitude gain coefficient. Constraint condition $C_{2}$ is used to control the gain value of the auxiliary beam less than the gain value of the main beam, and constraint condition $C_{3}$ is used to control the ratio of the auxiliary beam. Furthermore, $\varepsilon_{1}$ and $\varepsilon_{2}$ are the upper and lower limits of the sidelobe level, respectively.

Considering the small power gain difference that can be achieved by changing only Bayliss model parameters, the optimized performance of Model (12) will be verified by simulation in Section 4. Therefore, the auxiliary beam pointing optimization variable is added based on the above model. The optimized model can be represented as

(13) $$\begin{array}{l} \;\underset{\theta_{a},R,\lambda }{\max}\frac{C_{i}}{\sum_{\begin{array}{l} j>i\\ i\neq H_{m} \end{array}}C_{j}+N_{a}}.\\ s.t.\;C_{1}:\frac{C_{i}}{\sum_{\begin{array}{l} j>i\\ i\neq H_{m} \end{array}}C_{j}+N_{a}}>\delta \\ \;C_{2}:0<\lambda <\kappa \;(i<j,i\sim j\in Q),\\ \;C_{3}:\varepsilon_{1}<R<\varepsilon_{2}\\ \;C_{4}:-\beta <\theta_{a2}<\beta \end{array}$$

where β is the width of the main lobe of the main beam, and $\theta_{a2}$ is the moving angle of the auxiliary beam relative to the main beam.

It is assumed that the estimated SNR and noise power of the collision signal in the main channel are $\hat{\gamma }$ and ${\hat{N}}_{m}$, respectively. The carrier power of the collision signal in the main channel is written as

(14) $${\hat{C}}_{m}=\hat{\gamma }\cdot {\hat{N}}_{m}.$$

The directional diagram gain of the main beam is expressed as $G_{rm}\left(\theta ,\theta_{0}\right)={\left|W_{m}^{H}a\left(\theta \right)\right|}^{2}={\left|a{\left(\theta_{0}\right)}^{H}a\left(\theta \right)\right|}^{2}$, and the main beam direction is $\theta_{0}=0{}^{\circ}$. According to Equation (11), the gain of the auxiliary beam directional diagram can be described as $G_{ra2}\left(\theta ,\theta_{a2},\lambda ,R\right)={\left|\lambda \cdot {\left(a\left(\theta_{a2}\right)\cdot W_{Bayliss}^{H}(\bar{n},R,N)\right)}^{H}a\left(\theta \right)\right|}^{2}$. In this paper, the auxiliary beam designed is generated by the same antenna as the main beam. Therefore, the power and power gain ratio of the main channel and auxiliary channel are the same, that is, $C/G_{ra}\left(\theta ,\theta_{a},\lambda ,R\right)={\hat{C}}_{m}/G_{rm}\left(\theta ,\theta_{0}\right)$. Consequently, the carrier-to-noise ratio of the collision signal in the auxiliary channel is calculated with the help of the ratio of power to power gain. The signal power of the collision signal in the auxiliary channel is represented as $C=G_{ra}\left(\theta ,\theta_{a},\lambda ,R\right)\cdot \left({\hat{C}}_{m}/G_{rm}\left(\theta ,\theta_{0}\right)\right)$. As a result, the CINR of the collision signal in the auxiliary channel can be expressed as

(15) $$\begin{array}{l} \frac{C_{i}}{\sum_{\begin{array}{l} j>i\\ i\neq H_{m} \end{array}}C_{j}+{\hat{N}}_{a}}=\frac{G_{ra}\left(\theta_{i},\theta_{a},\lambda ,R\right)\cdot \left({\hat{C}}_{mi}/G_{rm}\left(\theta_{i},\theta_{0}\right)\right)}{\sum_{\begin{array}{l} j>i\\ i\neq H_{m} \end{array}}G_{ra}\left(\theta_{j},\theta_{a},\lambda ,R\right)\cdot \left({\hat{C}}_{mi}/G_{rm}\left(\theta_{j},\theta_{0}\right)\right)+{\hat{N}}_{a}}\\ \;=\frac{G_{ra}\left(\theta_{i},\theta_{a},\lambda ,R\right)\cdot \left({\hat{\gamma }}_{i}\cdot {\hat{N}}_{m}/G_{rm}\left(\theta_{i},\theta_{0}\right)\right)}{\sum_{\begin{array}{l} j>i\\ i\neq H_{m} \end{array}}G_{ra}\left(\theta_{j},\theta_{a},\lambda ,R\right)\cdot \left({\hat{\gamma }}_{j}\cdot {\hat{N}}_{m}/G_{rm}\left(\theta_{j},\theta_{0}\right)\right)+{\hat{N}}_{a}}, \end{array}$$

where ${\hat{N}}_{a}$ is the estimated noise power of the auxiliary channel, $G_{ra}\left(\theta_{i},\theta_{a},\lambda ,R\right)$ is the receiving gain of the ith collision signal with strong power in the auxiliary beam, and $G_{ra}\left(\theta_{j},\theta_{a},\lambda ,R\right)$ is the receiving gain of the jth collision signal with weak power in the auxiliary beam. In the bargain, $G_{rm}(\theta_{i},\theta_{0})$ is the receiving gain corresponding to the ith collision signal in the main beam, and $G_{rm}\left(\theta_{j},\theta_{0}\right)$ is the receiving gain corresponding to the jth collision signal in the main beam.

After the above derivation, the model of Equation (13) is transformed into

(16) $$\begin{array}{l} \;\underset{\theta_{a},R,\lambda }{\max}\frac{G_{ra2}\left(\theta_{i},\theta_{a2},\lambda ,R\right)\cdot \left({\hat{\gamma }}_{i}\cdot {\hat{N}}_{m}/G_{rm}\left(\theta_{i}\right)\right)}{\sum_{\begin{array}{l} j>i\\ i\neq H_{m} \end{array}}G_{ra2}\left(\theta_{j},\theta_{a2},\lambda ,R\right)\cdot \left({\hat{\gamma }}_{j}\cdot {\hat{N}}_{m}/G_{rm}\left(\theta_{j}\right)\right)+{\hat{N}}_{a}}.\\ s.t.\;C_{1}:\frac{G_{ra2}\left(\theta_{i},\theta_{a2},\lambda ,R\right)\cdot \left({\hat{\gamma }}_{i}\cdot {\hat{N}}_{m}/G_{rm}\left(\theta_{i}\right)\right)}{\sum_{\begin{array}{l} j>i\\ i\neq H_{m} \end{array}}G_{ra2}\left(\theta_{j},\theta_{a2},\lambda ,R\right)\cdot \left({\hat{\gamma }}_{j}\cdot {\hat{N}}_{m}/G_{rm}\left(\theta_{j}\right)\right)+{\hat{N}}_{a}}>\delta \\ \;C_{2}:0<\lambda <\kappa \;(i<j,i\sim j\in Q)\\ \;C_{3}:\varepsilon_{1}<R<\varepsilon_{2}\\ \;C_{4}:-\beta <\theta_{a2}<\beta \end{array}$$

### 3.3. Optimization Model Solution

It can be seen from Equation (16) that the objective function of the optimization model is the same as that of the first inequality constraint function. Therefore, the solution of the optimization model can be broken down into the solution of (17) and counting the success rate of whether or not Equation (18) meets the separation threshold as follows:

(17) $$\begin{array}{l} \underset{\theta_{a},R,\lambda }{\max}\frac{G_{ra2}\left(\theta_{i},\theta_{a2},\lambda ,R\right)\cdot \left({\hat{\gamma }}_{i}\cdot {\hat{N}}_{m}/G_{rm}\left(\theta_{i}\right)\right)}{\sum_{\begin{array}{l} j>i\\ i\neq H_{m} \end{array}}G_{ra2}\left(\theta_{j},\theta_{a2},\lambda ,R\right)\cdot \left({\hat{\gamma }}_{j}\cdot {\hat{N}}_{m}/G_{rm}\left(\theta_{j}\right)\right)+{\hat{N}}_{a}}.\\ s.t.\;C_{1}:0<\lambda <\kappa \;(i<j,i\sim j\in Q)\\ \;C_{2}:\varepsilon_{1}<R<\varepsilon_{2}\\ \;C_{3}:-\beta <\theta_{a2}<\beta \end{array}$$

(18) $$\frac{G_{ra2}\left(\theta_{i},\theta_{a2},\lambda ,R\right)\cdot \left({\hat{\gamma }}_{i}\cdot {\hat{N}}_{m}/G_{rm}\left(\theta_{i}\right)\right)}{\sum_{\begin{array}{l} j>i\\ i\neq H_{m} \end{array}}G_{ra2}\left(\theta_{j},\theta_{a2},\lambda ,R\right)\cdot \left({\hat{\gamma }}_{j}\cdot {\hat{N}}_{m}/G_{rm}\left(\theta_{j}\right)\right)+{\hat{N}}_{a}}>\delta .$$

**Figure 4:**
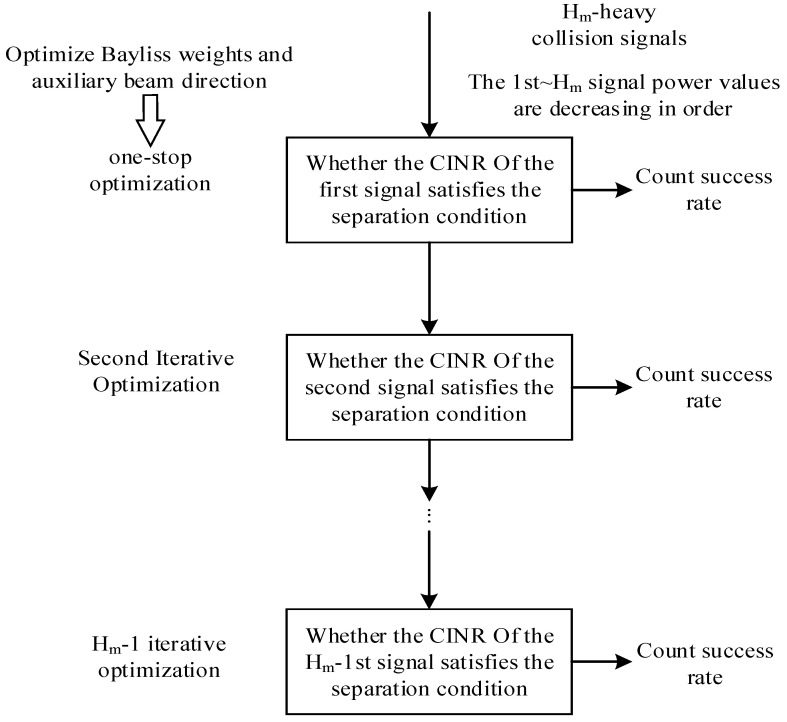
Flowchart for solving the auxiliary beam optimization model based on Bayliss window function weight optimization.

Formula (17) is a single-objective solution and multi-variable optimization problem, which creates separation conditions for packets colliding at a certain time slot. For the collision of multiple packets, it can be solved by iterative optimization step by step. The solution flow chart is shown in Figure 4.

The specific steps are as follows:

Step 1: Sequence the collision signals received in the auxiliary beam in accordance with the obtained power gain.

Step 2: Demodulate the first strong signal and take the maximum value of $\frac{C_{1}}{\sum_{j>1}C_{j}+{\hat{N}}_{a}}$ as the objective function. Two basic beam orientations, $\theta_{1}$ and $\theta_{2}$, are deemed as variables. After the optimal CINR is obtained, whether the separation threshold is reached is judged, and the success rate is counted.

Step 3: If the first strong signal can be demodulated successfully, continue demodulating the second strong signal for the remaining $H_{m}-1$ collision packets. Then, take the maximum value of $\frac{C_{2}}{\sum_{j>2}C_{j}+{\hat{N}}_{a}}$ as the objective function and obtain the optimal CINR by adjusting the basic beam orientations $\theta_{1}$ and $\theta_{2}$. Determine whether the separation threshold is reached and calculate the success rate. Repeat the above steps until the $H_{m}-1$ highest power signal is demodulated.

**Figure 5:**
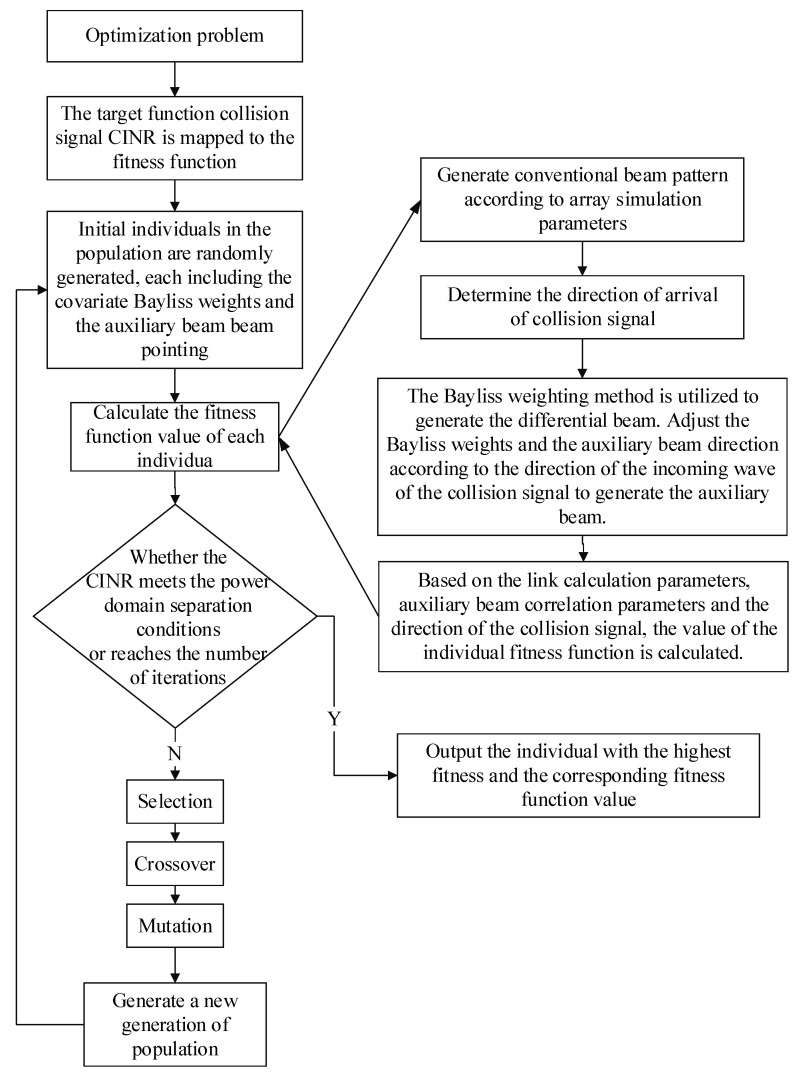
Genetic algorithm calculation process.

In this paper, the genetic algorithm (GA) is introduced several times to solve the auxiliary beam optimization model for the above solution process. GA exhibits favorable global optimization properties, demonstrates robust convergence, requires less computing time, and offers high robustness. The flow chart of the algorithm is shown in Figure 5.

## 4. Results

The simulation link parameters and array parameters of this scheme are shown in Table 3.

In order to verify the feasibility and performance of the auxiliary beam scheme based on Bayliss weight optimization, a simulation analysis is carried out from the perspectives of system access performance and error robustness.

### 4.1. System Access Performance Analysis

#### 4.1.1. Example of Collision Signal Separation

**Figure 6:**
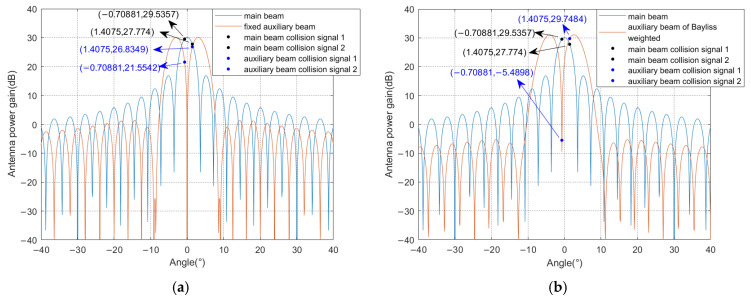
Example of collision signal separation for Bayliss weighted auxiliary beam scheme: (**a**) fixed auxiliary beam and (**b**) optimized auxiliary beam.

The parameters are set to 32 arrays, with the sidelobe level of −30 dB and the amplitude gain of 12.6109. The number of sidelobe adjacent to the main lobe of the expected constraint is 4, generating a fixed Bayliss weight. It is assumed that the DOA of the collision signal is −0.70881° and 1.4075°, respectively, and the direction of the fixed weight auxiliary beam facing Bayliss weighting is 0°. The sidelobe level, amplitude gain, and optimal direction of the auxiliary beam oriented to Bayliss weighting are calculated by GA as −40 dB, 13, and −0.71914°, respectively. The collision signal separation diagram for the Bayliss weighted auxiliary beam scheme is shown in Figure 6. It can be seen from the figure that the original collision signal power gain difference in the main beam is 1.7617 dB. Figure 6a shows that the power gain difference in the fixed auxiliary beam is 5.2807 dB, while Figure 6b shows that the power gain difference increases to 35.2382 dB in the optimized auxiliary beam. It follows that optimization of Bayliss weights while introducing the auxiliary beam pointing optimization can obtain a larger power gain difference.

#### 4.1.2. Access Performance Without Error

**Figure 7:**
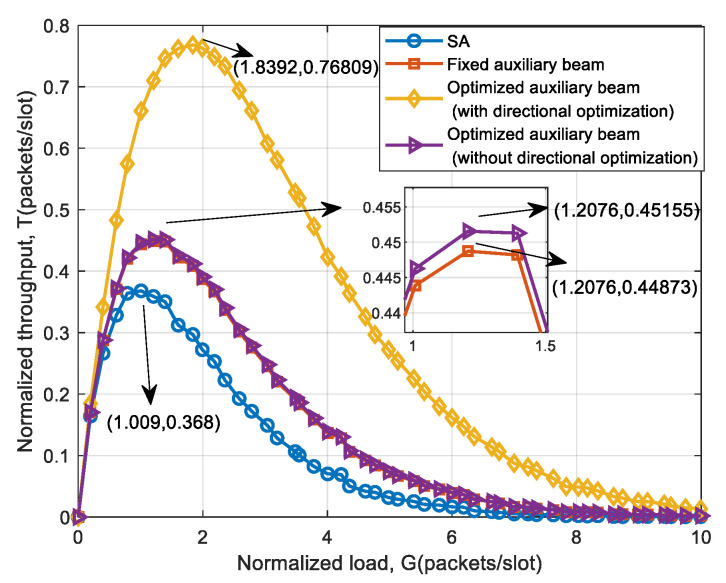
Throughput performance comparison of fixed, optimized auxiliary beam (with/without pointing).

To compare the system throughput performance of the auxiliary beam with a fixed weight, the auxiliary beam with parameters including directional optimization (refer to Model (12)), and the auxiliary beam with parameters that do not include directional optimization (refer to Model (13)), the three schemes are simulated respectively. The simulation results are shown in Figure 7.

As can be seen from Figure 7, the auxiliary beam with fixed Bayliss weight has the smallest system throughput, while the auxiliary beam with directional optimization has the largest system throughput. The throughput improvement of the three auxiliary beam schemes compared with SA is shown in Table 4. If only the Bayliss weight parameter is optimized, the system throughput is only increased by 0.76% compared to the fixed beam. It shows that the optimization space of Bayliss weight itself is small. Therefore, in Section 3, the auxiliary beam direction is used as an optimization parameter to obtain better system performance.

### 4.2. Error Robustness Analysis

In practical LEO satellite systems, attitude and orbit control maneuvers may degrade communication performance [27,28]. This paper models such effects as equivalent DOA errors for systematic analysis. Additionally, we account for array channel imperfections caused by inconsistent gain-phase responses among receiving antenna elements. Consequently, the effects of goniometric error and amplitude–phase error on the throughput and packet loss performance of the auxiliary beam access scheme are examined independently in this section. The array and error parameter settings are shown in Table 5.

#### 4.2.1. Estimation Error of DOA

**Figure 8:**
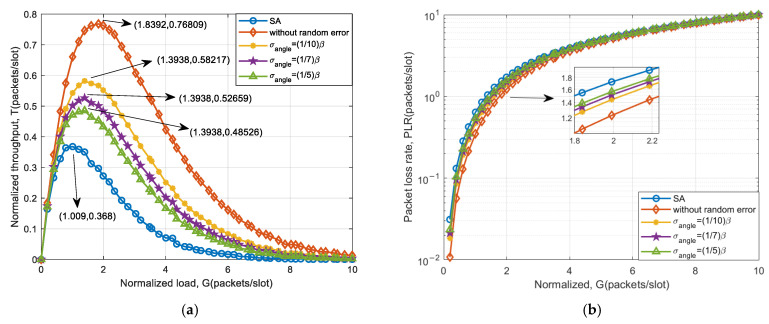
System access performance of an auxiliary beam-based scheme under 32-array goniometric error: (**a**) throughput and (**b**) packet loss rate.

Based on 32 elements, the system throughput and packet loss performance under angle measurement error are simulated. Moreover, the simulation results are compared with SA and the case without goniometric error. The simulation results are shown in Figure 8.

From Figure 8, it can be seen that the introduction of goniometric error significantly reduces the system throughput compared to accurate direction of arrival estimation. As the goniometric error increases, the system throughput decreases more, and the packet loss rate increases. However, when compared to the SA system, there remains a notable enhancement in throughput. This is attributed to the fact that goniometric errors may lead to signals that are potentially separable failing to fulfill the separation criteria, thereby decreasing the success rate of separating collision signals using auxiliary beam optimization schemes. Nevertheless, there is still a substantial probability that collision signals can be successfully separated and received. The system throughput improvement for different goniometric errors is shown in Table 6.

The results in Table 6 indicate that for a beamwidth of 3.2°, the peak throughput can be improved by more than 31.86% when the standard deviation of the estimation error of DOA is less than $\frac{1}{5}\beta$. For a beamwidth of 10.2°, the throughput peak can be increased by more than 28.24%. Therefore, the standard deviation σ that can be tolerated by both types of beams is an error of $\frac{1}{10}\beta ∼\frac{1}{5}\beta$. When the beamwidth increases, the throughput performance improvement slightly decreases. Due to the introduction of DOA estimation error, which is related to the main lobe width of the auxiliary beam, the magnitude of the error varies proportionally with the beamwidth. Different beamwidths have little effect on the improvement in system throughput performance.

#### 4.2.2. Estimation Error of Amplitude and Phase

**Figure 9:**
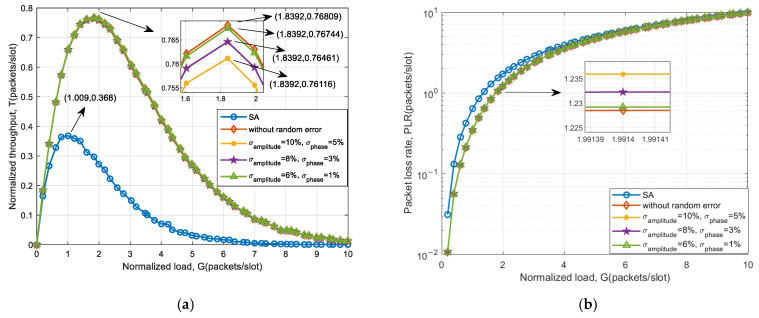
System access performance based on an auxiliary beam scheme under 32 array element amplitude–phase error: (**a**) throughput and (**b**) packet loss rate.

Figure 9 shows the simulation results of throughput and packet loss rate for a 32-element system. The comparison of system throughput improvement under different amplitude and phase errors is shown in Table 7.

The introduction of array amplitude–phase error has little effect on the success rate of separating collision signals. As can be seen from Figure 9, under the influence of amplitude and phase errors, the system throughput only decreases slightly compared with that without errors. Furthermore, as the introduced amplitude and phase error increase, the system throughput compared with SA shows a smaller increase while the system packet loss rate becomes larger. This is due to the fact that the array amplitude–phase error can also diminish the success rate of collision signal separation within this scheme. Nevertheless, only a small percentage of the collision signals cannot be received successfully, meaning that the amplitude–phase error has minimal effect on the performance of this scheme.

The throughput improvement is shown in Table 7. The results demonstrate that for a beamwidth of 3.2°, with a random phase error of 0.05 and an amplitude error of 0.1, the throughput peak can be enhanced by more than 106.84%. When the beamwidth is 10.2°, the peak throughput can be improved by more than 102.18%. As the beamwidth increases, the throughput performance improvement decreases. Since the introduced amplitude–phase error ultimately impacts the steering vector of the array, it results in an elevation of the sidelobes of the main beam, accompanied by a slight decrease in the gain peak. Furthermore, a smaller beamwidth results in a larger receive antenna gain peak and less gain peak drop. The above analysis shows that the scheme has a stronger tolerance for amplitude and phase error.

#### 4.2.3. Estimation Errors of DOA and Amplitude–Phase

**Figure 10:**
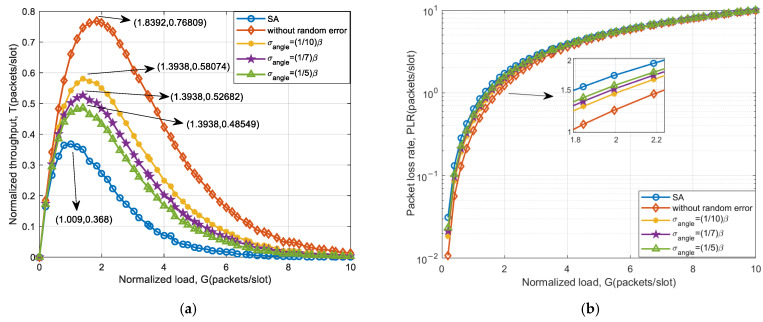
System access performance of an auxiliary beam-based scheme under 32-array goniometric error and amplitude-phase error: (**a**) throughput and (**b**) packet loss rate.

In this section, both goniometric error and amplitude–phase error are considered. The amplitude gain error follows a uniform distribution of [−0.1, 0.1], and the phase error follows a uniform distribution of [−0.05, 0.05]. For a system comprising 32 array elements, the success rate of separating collision signals using an auxiliary beam optimization access scheme is evaluated. Additionally, based on this success rate, simulations are conducted to assess the system’s throughput and packet loss rate performance. The simulation results are shown in Figure 10.

As observed in Figure 10, when amplitude–phase error and goniometric error are introduced simultaneously, the gain in throughput performance tends to decrease, and the rate of packet loss increases with the goniometric error. Additionally, the throughput improvement decreases with increasing beamwidth. The comparison of throughput improvement for different beamwidth simulation systems is shown in Table 8. For a beamwidth of 3.2° and considering $\sigma =\frac{1}{5}\beta$ simultaneously, the throughput improvement by introducing amplitude–phase error increased by 0.07% compared to using only goniometric error. This is due to the fact that the introduction of the gain and phase errors may offset the effect brought by a small part of the goniometric error. Consequently, the success rate of auxiliary beam separation of collision signals is slightly improved.

Combined with Table 6, Table 7 and Table 8, from the perspective of error influence, the throughput performance of goniometric error decreases more significantly than that of amplitude–phase error. The emergence of this issue stems from the fact that the estimation error associated with DOA represents a form of measurement error, whereas amplitude and phase errors within the array element channels constitute systematic errors. The introduction value of angle measurement error is larger than that of amplitude and phase error. Moreover, the amplitude–phase errors have a relatively small impact on the main lobe gain of the beam but a significant impact on the side lobe gain of the beam. Therefore, goniometric error has a greater impact on throughput. From the perspective of beamwidth, as the beamwidth increases, the basic beam roll-off band corresponding to 10 elements is smoother compared to 32 elements. As a consequence, flatter roll-off bands are generated within the main lobe of the auxiliary beam using Bayliss weighting. Due to the decrease in the peak gain of the auxiliary beam receiving, the success rate of the auxiliary beam separating collision signals with larger main lobe width decreases. Consequently, the system throughput decreases.

## 5. Conclusions

Building on previous research, this paper increases the complexity of auxiliary beam design and proposes a scheme featuring main lobe suppression. The scheme adopts the Bayliss-based differential beam weighting method. The auxiliary beam is generated from this differential beam, which makes the center of the master lobe produce zero trap and expands the power gain difference range of the collision signal. Based on the auxiliary beam pointing scheme, the success rate of collision signal separation is further improved so as to improve the overall throughput of the system. Simulation results show that compared with time slot ALOHA, the peak system throughput of this scheme increases by about 108%. The effect of the proposed scheme is obviously better than that of the optimization scheme based on auxiliary beam pointing. Furthermore, the scheme exhibits strong robustness, tolerating simultaneous systematic and measurement errors, making it a viable solution for large-scale random access in satellite IoT networks. Although the proposed beam design improves throughput, its static nulling pattern limits performance under dynamic interference. An intelligent beam-shaping framework that learns collision signal directions in real time could be a promising approach to unlock additional capacity gains.

## Figures and Tables

**Table 1:** The fourth-order polynomial coefficients and sidelobe levels of Bayliss directional pattern.

Polynomial Coefficients	Sidelobe Level/dB					
	−15	−20	−25	−30	−35	−40
A	1.0079	1.2247	1.4355	1.6413	1.8431	2.0415
$\Omega_{1}$	1.5124	1.6962	1.8826	2.0708	2.2602	2.4504
$\Omega_{2}$	2.2561	2.3698	2.4943	2.6275	2.7675	2.9123
$\Omega_{3}$	3.1693	3.2473	3.3351	3.4314	3.5352	3.6452
$\Omega_{4}$	4.1264	4.1854	4.2527	4.3276	4.4093	4.4973

**Table 2:** Polynomial coefficients $C_{n}$ of Bayliss directional diagram.

R	$C_{0}$	$C_{1}$	$C_{2}$	$C_{3}$	$C_{4}$
A	0.30387530	−0.05042922	−0.00027989	−0.00000343	−0.0000002
$\Omega_{1}$	0.98583020	−0.03338850	0.00014064	0.00000190	0.0000001
$\Omega_{2}$	2.00337487	−0.01141548	0.00041590	0.00000373	0.0000001
$\Omega_{3}$	3.00636321	−0.00683394	0.00029281	0.00000161	0.0000000
$\Omega_{4}$	4.00518423	−0.00501795	0.00021735	0.00000088	0.0000000

**Table 3:** Simulation parameters.

Parameter	Value
Carrier frequency (GHz)	2
Distance from satellite to Earth (km)	1000
Terminal transmission gain (dBi)	0
Bandwidth (kHz)	20
Interval of array elements (m)	wavelength/2
Terminal transmission power (dBW)	−10
Equivalent noise temperature (K)	290
Separation threshold (dB)	10

**Table 4:** The throughput improvement of fixed and optimized auxiliary beam (with/without pointing).

Auxiliary Beam Type	System Throughput Improvement
Fixed auxiliary beam	21.94%
Optimized auxiliary beam (without directional optimization)	22.70%
Optimized auxiliary beam (with directional optimization)	108.72%

**Table 5:** Array parameters and error settings.

Number of Array Elements	Beamwidth	DOA of Collision Signal	Estimation Error of DOA	Amplitude–Phase Error
32	3.2°	Random value of [−1.6°, 1.6°]	Obey a normal distribution with a mean of 0 and a standard deviation σ$\;\mathrm{of}\;\frac{1}{10}\beta$ $\mathrm{to}\;\frac{1}{5}\beta$	The phase error of the array element channel follows a uniformly distributed random value of 0.01–0.05, and the amplitude gain error of the array element follows a uniformly distributed random value of 0.06–0.1
10	10.2°	Random value of [−5.1°, 5.1°]		

**Table 6:** System throughput improvement under goniometric error.

Beamwidth	System Throughput Improvement		
	Without Error	$\sigma =\frac{1}{10}\beta$	$\sigma =\frac{1}{5}\beta$
3.2°	108.72%	58.35%	31.86%
10.2°	106.93%	55.28%	28.24%

**Table 7:** System throughput improvement under amplitude–phase error.

Amplitude–Phase Error	System Throughput Improvement	
Without error	108.72%	106.93%
$\sigma_{amplitude}=6\%,\sigma_{phase}=1\%$	108.54%	106.87%
$\sigma_{amplitude}=10\%,\sigma_{phase}=5\%$	106.84%	102.18%

**Table 8:** System throughput improvement under goniometric error and amplitude–phase error.

Beamwidth	System Throughput Improvement		
	Without Error	$\sigma =\frac{1}{10}\beta$	$\sigma =\frac{1}{5}\beta$
3.2°	108.72%	57.81%	31.93%
10.2°	106.93%	53.74%	27.86%

## Data Availability

The original contributions presented in this study are included in the article. Further inquiries can be directed to the corresponding author.
